# Taking practical learning in STEM education home: Examples from do‐it‐yourself experiments in plant biology

**DOI:** 10.1002/ece3.7207

**Published:** 2021-02-03

**Authors:** Ragnhild Gya, Anne Elisabeth Bjune

**Affiliations:** ^1^ Department of Biological Sciences Bjerknes Center for Climate Research University of Bergen Bergen Norway

**Keywords:** at‐home experiment, botany experiment, inquiry‐based learning, remote learning, student active

## Abstract

Practical teaching can give authentic learning experiences and teach valuable skills for undergraduate students in the STEM disciplines. One of the main ways of giving students such experiences, laboratory teaching, is met with many challenges such as budget cuts, increased use of virtual learning, and currently the university lockdowns due to the COVID‐19 pandemic. We highlight how at‐home do‐it‐yourself (DIY) experiments can be a good way to include physical interaction with your study organism, system, or technique to give the students a practical, authentic learning experience. We hope that by outlining the benefits of a practical, at‐home, DIY experiment we can inspire more people to design these teaching activities in the current remote teaching situation and beyond. By contributing two examples in the field of plant biology we enrich the database on experiments to draw inspiration from for these teaching methods.

## Graphical abstract

Practical teaching is met with many challenges, like budget cuts, moving to virtual teaching, and the lockdowns of the COVID pandemic. We highlight at‐home do‐it‐yourself experiments as a good option for maintaining hands‐on, student active, inquiry‐based teaching options when we teach off‐campus. We present two examples of such experiments from an undergraduate biology education in Norway.

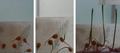

## 1 EXPERIENTIAL LEARNING OFF‐CAMPUS

Practice and practical activities in education provide considerable positive influence on the learning and motivation of students (Brownell et al., 2012; Easton & Gilburn, 2012; Hole, 2017; Lave, 1996; White et al., 2002). Practical learning experiences provide opportunities to engage multiple senses as you touch, smell and observe a study object or phenomenon, which creates a new way of knowing the theory by increased sensory and cognitive activity (Nabors et al., 2009; Willis, 2007). By including both vision, hearing, touch, and smell the students can link the knowledge to several parts of the brain, and this makes it easier to find that knowledge again later (Willis, 2007). Teaching in ways that include several senses, such as practical activities and experiments can therefore increase the learning outcomes for students (Nabors et al., 2009; Willis, 2007).

The positive effects of practical learning all depend on the pedagogical quality of the activities, and how the students process the new knowledge they obtain through this teaching. Specifically, student engagement is important for the learning outcome of the practical activity. On one hand, there are the “cookbook” laboratories where students do not engage in any of the decision‐making and just perform activities, more or less, mindlessly following the protocol. If students only follow a “cookbook recipe” without being engaged in the learning situation, they will not gain the same outputs as students who are more involved in the decision‐making of the learning activities (Brownell et al., 2012). On the other hand, in more student‐active practical work, activities can give the students skills and competencies related more to an authentic science experience and thus better prepare the students for work‐life (Hole, 2017). Practical learning can be active and authentic in many ways, from the process of critical thinking and generating hypotheses to designing the experiment and analyzing the collected data (Spell et al., 2014). In the most student‐active end of the spectrum we find highly authentic research experiences where the students pose novel questions, find new results thereby making new discoveries to the scientific field, and even publish the data (Aikens, 2020; Ballen et al., 2017; Rodenbusch et al., 2016; Spell et al., 2014). Implementing all these aspects is not possible in all courses, but most courses can plan their experiments as a student‐led inquiry‐based activity by engaging the students in planning the hypothesis and tailor parts of the experiment to these hypotheses (Ballen et al., 2017). Through student‐led inquiry‐based experiments, students achieve better learning (Hilfert‐Rüppell et al., 2013), especially the least prepared students (Blumer & Beck, 2019), and they gain a higher academic self‐esteem, which is associated with higher rates of retention (Aikens, 2020; Brownell et al., 2012; Gormally et al., 2009; Harrison et al., 2011; Hofferber et al., 2016). Seeing all these benefits, experiments in all courses should aim to engage the students by facilitating autonomy in the learning situation.

Knowing the benefits of student engagement and hands‐on experiments it is very unfortunate that practical activities are currently facing several challenges. All over the world, there are less practical in‐person learning activities for undergraduate students because of one or more of these challenges; budget cuts (Baker & Verran, 2004; Hearing & Lu, 2014), increased use of technology (Jones, 2018), and the largest, most acute challenge; the ongoing COVID‐19 pandemic (Campbell et al., 2020; Sahu, 2020). During the COVID‐19 pandemic many universities all over the world were, and still are, faced with closed facilities (Bao, 2020; Sahu, 2020). Practical teaching had to be re‐designed to fit off‐campus teaching which had consequences for many important educational platforms in the STEM (Science, Technology, Engineering, and Mathematics) disciplines, such as laboratory training (Campbell et al., 2020; Noel et al., 2020), clinical laboratories (Cai et al., 2020; Franchi, 2020), and field courses (Barton, 2020; Creech & Shriner, 2020).

When our teaching laboratories are shut down and we no longer can meet the students in person, how do we meet the intended learning outcomes from the planned practical teaching? One main challenge for student learning outcomes when transferring from on‐campus to online teaching, is to include inquiry‐based active learning, and let students apply their knowledge in the virtual setting (Hines et al., 2020). Virtual laboratories are one option, and these already exist for some STEM disciplines such as microbiology (Makransky et al., 2019), human anatomy (Sorgo et al., 2008), molecular biology (White et al., 2002), physics (Finkelstein et al., 2005; Olympiou & Zacharia, 2012), chemistry (Guarracino, 2020), and ecology (Wu et al., 2016). Virtual laboratories can be a good option for off‐campus teaching, as they seem to give students the same learning outcomes if they do them at home, as in class with teacher supervision (Faulconer & Gruss, 2018; Makransky et al., 2019). Virtual labs are found to increase the learning outcomes by giving students the option to repeat experiments as much as needed (Baker & Verran, 2004), practice before the in‐person laboratory (Moreno‐Ger et al., 2010), and learning about experimental design (Darius et al., 2007). For very abstract and complex phenomena such as the electron movement in electricity (Finkelstein et al., 2005), or light and color (Olympiou & Zacharia, 2012) the option of redoing and choosing speed in the virtual laboratories can give better learning outcomes for the students than traditional laboratories. However, for less complex phenomena the best use of virtual laboratories for student learning outcomes is when it is used in combination with lectures and in‐person laboratories (Baker & Verran, 2004; Brockman et al., 2020; de Jong et al., 2013). The virtual laboratories help students draw the link between the theory from lectures, and the practice from the in‐person laboratory (de Jong et al., 2013). When virtual laboratories are a stand‐alone option, the learning outcomes are not as good compared with an in‐person laboratory where the student can interact with the study specimen, tools, or technics (Noel et al., 2020; Peat & Taylor, 2005). In addition, there are certain skills and laboratory techniques that students are expected to know at the end of a degree, which cannot be taught through virtual laboratories alone (Brinson, 2015; Noel et al., 2020). Thus, replacing all in‐person labs with virtual laboratories, for example due to COVID‐19 campus lockdown, could negatively affect student learning outcomes in STEM subjects.

A better option could be at‐home, do‐it‐yourself (DIY) experiments that incorporates the physical interaction with the study organism, technique, or system of interest, which could be relevant in many fields within the STEM disciplines. At‐home DIY experiments can support and increase student motivation and assure course alignment by obtaining learning objectives on both theoretical knowledge, and practical skills. Not only do these kinds of experiments give students similar learning outcomes as an in‐person laboratory, but they also give students the opportunity to do it in their own pace and redoing if needed, which increases the students motivation for the subject and interest for science (Gendjova, 2007; Zurlifan et al., 2018). Combining the increased autonomy by allowing students to do the experiments by themselves, with an inquiry‐based experimental design will increase students' academic self‐esteem (Ballen et al., 2017; Zurlifan et al., 2018). However, as with all off‐campus teaching, the lack of in‐person interaction between student and teacher, as well as student‐student collaborations, could have negative effects on the students learning outcome (Faulconer & Gruss, 2018; Theodosiou & Corbin, 2020) and social connections with the group (Creech & Shriner, 2020; Theodosiou & Corbin, 2020).

During the pandemic lockdowns, many instructors managed to maintain the practical experience and skills training by designing at‐home DIY experiments. Some sent equipment to students, others designed an experiment using only things all students had at home (Fox et al., 2020; Shivam & Wagoner, 2020). There are some laboratory kit options (such as eScience^1^) that can be purchased and sent to students, but course budgets might be a restriction. For experiments using equipment, students have at home, we need to have student equity in mind (Barton, 2020; Creech & Shriner, 2020; Fox et al., 2020), especially if it requires high‐cost equipment like computers, smartphones, or specific software, but also with everyday things like batteries, magnets etc. (Fox et al., 2020). Designing such experiments requires flexibility enough to adapt to all the different students home situations for all to be able to achieve the same learning outcomes. To our knowledge, there are few examples of such at‐home DIY experiments in STEM disciplines documented in the literature, but we found some in physics (Fox et al., 2020; Turner & Parisi, 2008), engineering (Rossiter et al., 2019) chemistry (Gendjova, 2007), and ecology (Creech & Shriner, 2020).

Here we report on two at‐home, DIY, inquiry‐based laboratory experiments in plant biology. Our experiments are designed to use equipment easily available for all students, and plants that can be found anywhere outside no matter where the students live. In the first of these experiments, students find the water‐holding capacity of different kinds of bryophytes, in the second, students germinate seeds to observe and follow the seedling development.

## 2 BIO101 AND THE COVID‐19 LOCKDOWN

The laboratory course described here is a module of an evolution and systematics course (BIO101, Organismal Biology I^2^) for first‐year bachelor degree students at the University of Bergen (Norway). In this course, the students learn about evolutionary development and adaptations, and taxonomy in three different modules: microbiology, zoology, and botany. This is a practical course including a laboratory in each of the three modules. The botany module covers the evolutionary development of plants, the current systematics, and morphological differences between groups. We divide the topics of the laboratory days into the plant groups; bryophytes, ferns and lycopods, gymnosperms, and angiosperms.

During the spring term 2020, the University of Bergen, as many other universities in the world (Sahu, 2020), shut down all on‐campus teaching. The entire botany module (lectures, seminars, and laboratory work) was moved online on very short notice. To meet the intended learning outcomes for the students when we could not proceed with our planned laboratory course, we chose to give the students two at‐home DIY experiments as part of the online teaching. Our aim was to give our students physical tasks to remain engaged and motivated despite the abrupt transition to remote instruction. The two experiments were a test of water‐holding capacity in bryophytes and a seed germination trial. By sharing these at‐home DIY experiments we increase the number of described experiments of this kind in the STEM disciplines, for people to use, or draw inspiration from.

## 3 WATER‐HOLDING CAPACITY IN BRYOPHYTES

One of the main intended learning outcomes of this course is to know how plants have evolved different strategies to meet the challenges of growing on land, specifically the risk of drying out and reproduction in a non‐liquid medium. We designed this experiment to illustrate how bryophytes with different morphological structures and adaptations have different water‐holding capacities. The morphological structures for holding and storing water in bryophyte, range from lamellae, and hyaline cells, to the structure of the branching in the bryophyte (Smith, 2012). Different species and genera have specific morphological structures, and ecological strategies for avoiding drying out. In this experiment, we test how the species and their strategies vary in their ability to store water.

**FIGURE 1:**
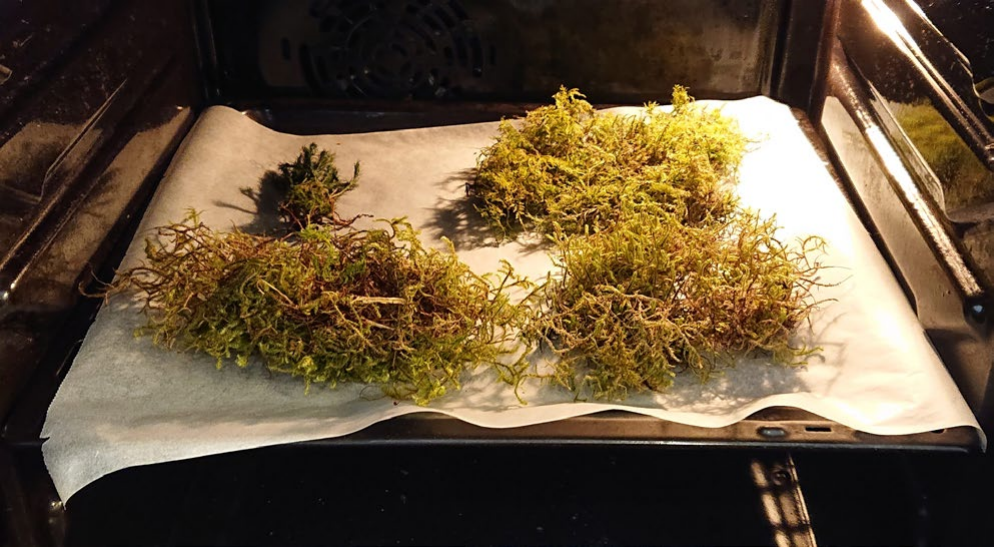
Drying four different species of bryophytes in a regular oven at 40°C for a couple of hours to obtain dry weight. This is step one in a DIY at‐home experiment to find the water holding capacity of different kinds of bryophytes. Photo: Ragnhild Gya

The students decided what they wanted to test and formed hypotheses before they collected at least two different types of bryophytes, depending on the hypothesis. The students came up with many good hypotheses, for example, testing morphology (i.e., many branches vs. non‐branched bryophytes), or habitats (i.e., on a rock vs. in the riverbank). We provided the students with a simple bryophyte identification tool for the most common bryophytes in the area.^1^ This way the students could identify some species or at least the genus on their own and could use this in the hypothesis testing. When the students conducted the experiment, they followed the protocol that we provided for them (Appendix 1). This involved drying the bryophytes completely either over several days at air temperature, or in an oven (Figure 1), and weighing the dry weight of the collected bryophytes on a regular kitchen scale. To get the amount of water the bryophyte can store the students soaked the bryophytes completely and then weighed it again when it was saturated with water. The difference between the dry weight and the wet weight gave us the answer to how much water the bryophyte can hold. After finishing the experiment, the students wrote a report including their hypothesis, results, and a short discussion of what their results indicated, linking back to their hypothesis (the protocol in Appendix 1 gives an outline for what the students included in the report).

The data on bryophyte dry and wet weights was shared in an online form so that everyone in the class could access all the data. These data were used in the seminars as discussion points for broader patterns across species, and they were also available for the students to include in their report if they wanted to expand the data material to better answer their hypothesis. As a group, we found out that most of the bryophytes with high water‐holding capacity were from the genus *Sphagnum*, which have hyaline cells for storing water. In total the class collected more than 15 different genera of bryophytes.

## 4 SEED GERMINATION EXPERIMENT

Another important part of the intended learning outcomes in this course, is to know the difference between the two main groups of flowering plants (angiosperms)—the monocotyledons and the dicotyledons. This experiment gives the students some real‐life experience with two of the important characters (root systems architecture and number of cotyledons). The seed germination experiment is easily conducted at home with few resources and gives a solid base for discussing characters used to differentiate between the two groups. The students followed a protocol we prepared for them also in this experiment (Appendix 2).

**FIGURE 2:**
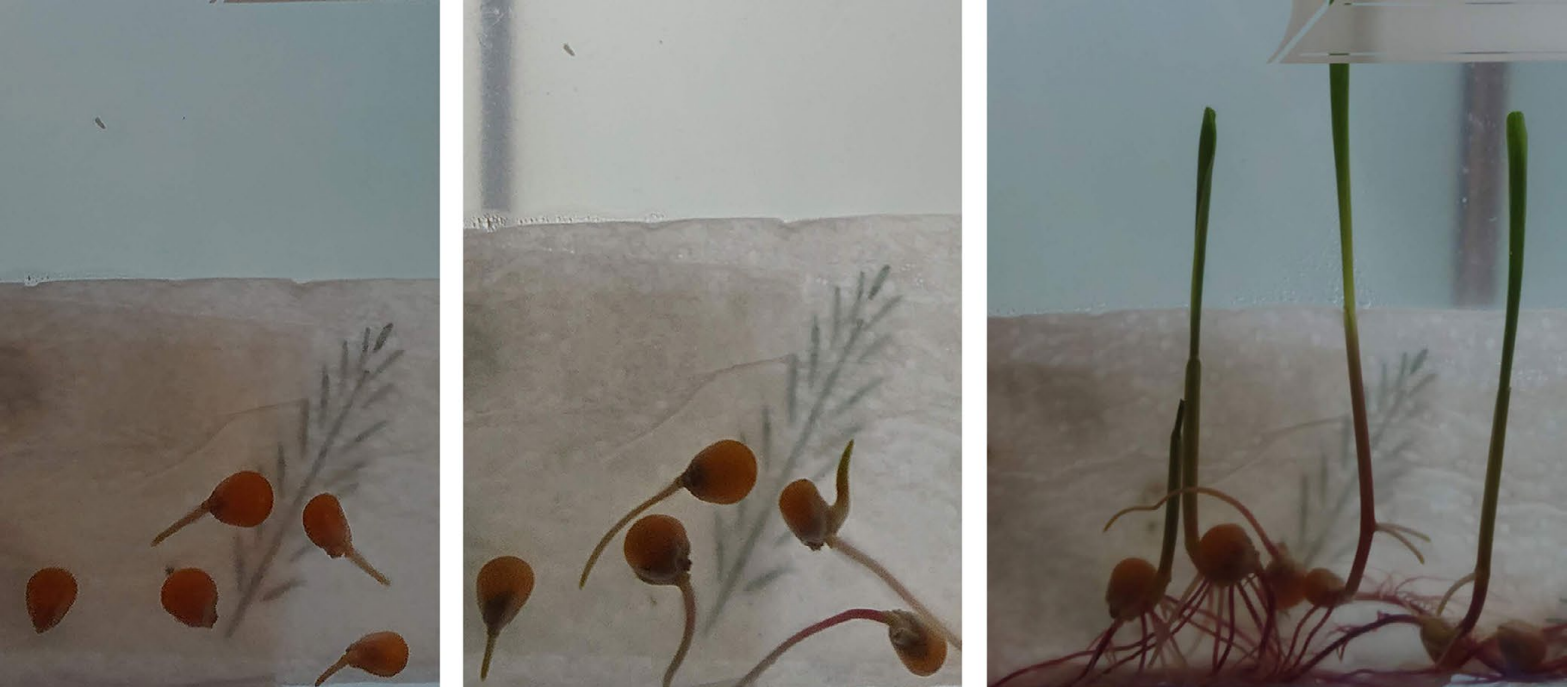
Three times during a seed germination experiment conducted by students at home during COVID‐19 lock down. This is corn seeds and their germination after 3 days, 5 days, and 11 days. Photo: Ragnhild Gya

The students found at least two different kinds of seeds at home to conduct this experiment. The seeds could be from fresh fruits such as apples or tomatoes, or they could use dry seeds that many people have in their kitchen drawers, like chia or linseeds. To provide the best growing conditions, the students put the seeds on moist paper towels in a sealed plastic bag and hung them on a window with plenty of light and warm conditions. The students documented the experiment with a short report including photographs of the seed development taken twice a week (Figure 2). They wrote a short text arguing for whether the species they sowed were a monocotyledon or a dicotyledon. The observations during the experiment of the characters distinguishing the two groups formed the basis of the conclusion in the report which the students submitted from the experiment.

In the submitted reports we saw that the students grew many different kinds of seeds; including corn, apple, cucumber, tomato, quinoa, pea, sunflower, pumpkin, paprika, and even avocado (this had been developing for a long time before we started). Many of the students told us that they had transferred the seeds to soil and wanted to keep the plants growing over summer.

## 5 OUTCOME OF THE TWO EXPERIMENTS

Here we have outlined two DIY experiments that can easily be conducted from home. We designed the water‐holding capacity experiment for the virtual laboratory during the COVID lockdown in 2020 and redesigned the germination experiment that we had used in previous years. When we conducted the germination experiment previously, we gave students both a monocotyledon species and a dicotyledon species. In 2020, the students chose what to grow themselves which gave the experiment a more student active inquiry‐based component. When we compare the student reports submitted in 2019 and 2020 from the germination experiment, we see that in 2019 there were 57% (*n* = 60) of the students that had a full score, while in 2020, 90% (*n* = 61) of the students did. Student scores were higher, and the discussion parts of the reports were more curiosity driven for the 2020 students (personal observations). The students expressed appreciation and motivation for both experiments, as they offered an opportunity for engaging in a practical activity after weeks on lockdown with video lectures as the only learning activity.

The changes made to the practical teaching in the spring term of 2020 was done in a hurry because of the sudden lockdown. With more time to plan, it would be possible to plan the laboratory activities better and make learning outcomes for the students even better. Specifically, linking the experiments with the lectures and discussion seminars in a better way. In a situation with only off‐campus teaching, it could be an advantage to increase interaction between students and teachers, and facilitating the social aspect of learning through more collaboration between students (Faulconer & Gruss, 2018). When we have more time to plan for what will likely be partially off‐campus semesters in the near future, we strongly recommend proper planning of the course alignment from intended learning outcomes, through teaching material and methods, to testing of learning outcomes.

## 6 CONCLUSIONS

Both experiments presented in this paper are designed to include some autonomy, either through hypothesis formulation and planning of data collection, or choice of what species to use for the germination test—making them student‐led inquiry‐based experiments (Ballen et al., 2017). The combination of being able to do something active and practical in the mostly virtual teaching environment, and the autonomy of choosing the hypothesis and study organism themselves could explain the perceived high motivation of the students (Hofferber et al., 2016). We think that experiments like these will become a valuable asset to on‐campus teaching as well as partially and fully off‐campus teaching. Personally, we as instructors had some very positive experiences with these two at‐home DIY experiments, and plan to incorporate both experiments in the same way next year, even if the rest of the laboratory course goes back to normal on‐campus teaching. We hope that sharing these experiments, along with our experiences, can be helpful as a tool for other instructors to include more student‐led inquiry‐based activities in laboratory courses both on‐campus and off‐campus.

## CONFLICT OF INTEREST

None declared.

## AUTHOR CONTRIBUTIONS

**Ragnhild Gya:** Conceptualization (equal), methodology (lead), visualization (lead), writing‐original draft (lead), writing: review and editing (equal). **Anne Elisabeth Bjune:** Conceptualization (equal), methodology (supporting), visualization (supporting), writing: original draft (supporting), writing: review and editing (equal).

## Supporting information

Appendix S1

Appendix S2

## ENDNOTES

^2^
https://www.uib.no/en/course/BIO101.

^1^ https://esciencelabs.com/

^2^ https://www.uib.no/en/course/BIO101

## Appendix 1

### Water‐holding capacity experiment template

#### Water holding capacity

Many mosses are extremely capable of holding and storing water many times their own weight. In the experiment, you have done at home you have tested how much the moss weighs when it is dry and how much water it can hold. You should enter the data from your experiment using this link (link to a google drive document or similar where students can share the data).

If you are unable to collect your own moss, you can select some species from the list in the shared data document (link to google drive document) and use those data in the discussion below.

**The hypothesis you want to test are:**

**Photographs of the moss (es) when they are dry:**

**Photographs of the moss (es) when they are soaked:**

**Example of table for entering data:**

**Table:**

	Moss 1	Moss 2
Dry weight moss (g)		
Wet weight moss(g)		
Amount of water (g)		
Ratio water/dry weight moss (g)		

**What conclusions can you draw from your own results?**

## Appendix 2

### Seed germination experiment template

#### Seed germination experiment

Find some seeds that you have in your kitchen drawer or fridge and see how they develop as they germinate. We have tested corn (as in popcorn), linseeds, chia seeds, yellow peas, pumpkin and, sunflower seeds from the kitchen drawer and blueberries, tomatoes, red peppers, chili, and peas from the fridge. There are many more types you can use.

The aim of this experiment is that you should observe how seeds germinate, and you should be able to classify your species depending on how it germinates.

**Before you start**

**Which specie(s) are you going to use?**

**Insert photographs of the seeds**

#### Starting the experiment

Take kitchen paper and add some water to it before you place it in a transparent plastic bag. Place the seeds on the wet kitchen paper, blow some air into the bag, and seal it. Use one bag per type of seed that you are going to observe. The best is to use a zip lock bag, but any transparent bag that you can tie will do. It is important that the bag is completely tight.

Hang your bag(s) with the seed(s) in a window or another light and warm spot.

#### Observations along the germination

You should observe your seeds at least twice a week. The best is to set 2 days a week—such as Monday and Thursday. Take a picture of the development on those days. If it makes it easier you can open the bag(s) to take the photograph, just remember to lock it afterward. For your report choose four photographs that show the development from start to end. Remember to insert the date when you took the photograph. Try to take the photograph so that you show the root, the stem, and the leaves of the germinating plant. Ideally, you should compare the minimum two species (one monocot and one dicot). You can work together in your groups and share images.

**To classify your plants you should observe and report on the following:**

**How many cotyledons are emerging?**

**How would you describe the cotyledons? You can use drawings and photographs if you want.**

**What about the root? Is there one main root or is the root system fibrous without a main root?**

**Based on your observations and photographs of the germination ‐ are your plants monocots or dicots? And why?**
