# Supplementary material for: Taking practical learning in STEM education home: Examples from do‐it‐yourself experiments in plant biology
Source: Ecol Evol. 2021 Feb 3;11(8):3481–7. doi: 10.1002/ece3.7207 (PMC8057327; doi:10.1002/ece3.7207)
Supplement: Supplementary file 1 — Appendix S1 [file ECE3-11-3481-s002.docx]

**Appendix 1 - Water holding capacity experiment template**

**Water holding capacity**

Many mosses are extremely capable of holding and storing water many times their own weight. In the experiment you have done at home you have tested how much the moss weighs when it is dry and how much water it can hold. You should enter the data from your experiment using this link (link to a google drive document or similar where students can share the data).

If you are unable to collect your own moss, you can select some species from the list in the shared data document (link to google drive document) and use those data in the discussion below.

**The hypothesis you want to test are:**

**Photos of the moss(es) when they are dry:**

**Photos of the moss(es) when they are soaked:**

**Example of table for entering data:**

|  | **Moss 1** | **Moss 2** |
| --- | --- | --- |
| **Dry weight moss (g)** |  |  |
| **Wet weight moss(g)** |  |  |
| **Amount of water (g)** |  |  |
| **Ratio water/dry weight moss (g)** |  |  |

**What conclusions can you draw from your own results?**
