# Supplementary material for: Taking practical learning in STEM education home: Examples from do‐it‐yourself experiments in plant biology
Source: Ecol Evol. 2021 Feb 3;11(8):3481–7. doi: 10.1002/ece3.7207 (PMC8057327; doi:10.1002/ece3.7207)
Supplement: Supplementary file 2 — Appendix S2 [file ECE3-11-3481-s001.docx]

**Appendix 2 - Seed germination experiment template**

**Seed germination experiment**

Find some seeds that you have in your kitchen drawer or fridge and see how they develop as they germinate. We have tested corn (as in popcorn), linseeds, chia seeds, yellow peas, pumpkin and sunflower seeds from the kitchen drawer and blueberries, tomatoes, red peppers, chili and peas from the fridge. There are many more types you can use.

The aim of this experiment is that you should observe how seeds germinate and you should be able to classify your species depending on how it germinates.

**Before you start**

**Which specie(s) are you going to use?**

**Insert photos of the seeds**

**Starting the experiment**

Take kitchen paper and add some water to it before you place it in a transparent plastic bag. Place the seeds on the wet kitchen paper, blow some air into the bag, and seal it. Use one bag per type of seed that you are going to observe. The best is to use a zip lock bag, but any transparent bag that you can tie will do. It is important that the bag is completely tight.

Hang your bag(s) with the seed(s) in a window or another light and warm spot.

**Observations along the germination**

You should observe your seeds at least twice a week. The best is to set two days a week - such as Monday and Thursday. Take a picture of the development on those days. If it makes it easier you can open the bag(s) to take the photo, just remember to lock it afterwards. For your report choose four photos that show the development from start to end. Remember to insert the date when you took the photo. Try to take the photo so that you show the root, the stem, and the leaves of the germinating plant. Ideally you should compare the minimum two species (one monocot and one dicot). You can work together in your groups and share images.

**To classify your plants you should observe and report on the following:**

**How many cotyledons are emerging?**

**How would you describe the cotyledons? You can use drawings and photos if you want.**

**What about the root? Is there one main root or is the root system fibrous without a main root?**

**Based on your observations and photos of the germination - are your plants monocots or dicots? And why?**
